# Supplementary material for: Thioredoxin-interacting protein regulates protein disulfide isomerases and endoplasmic reticulum stress
Source: EMBO Mol Med. 2014 May 19;6(6):732–43. doi: 10.15252/emmm.201302561 (PMC4203352; doi:10.15252/emmm.201302561)
Supplement: Supplementary file 1 — Supplementary Figure S1 [file emmm0006-0732-sd1.pdf]

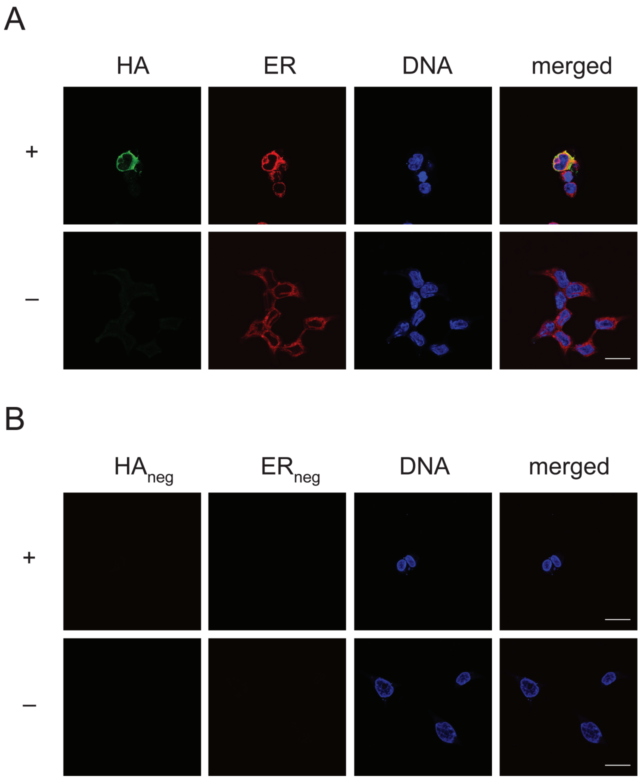

Supplementary Figure S1. HA-tagged PDI is localized in the ER. HEK293TN cells were transfected with HA-tagged PDI (+) or an empty vector (–). Cells were subsequently fixed, permeabilized and stained for HA or ERp72 (ER). Scale bar = 25 µm. **A.** Protein levels of HA-tagged PDI and ER marker ERp72 visualized by immunofluorescence under confocal microscopy with **B.** negative controls, probed with secondary antibodies only.
