# Supplementary material for: Thioredoxin-interacting protein regulates protein disulfide isomerases and endoplasmic reticulum stress
Source: EMBO Mol Med. 2014 May 19;6(6):732–43. doi: 10.15252/emmm.201302561 (PMC4203352; doi:10.15252/emmm.201302561)
Supplement: Supplementary file 2 — Supplementary Figure S2 [file emmm0006-0732-sd2.pdf]

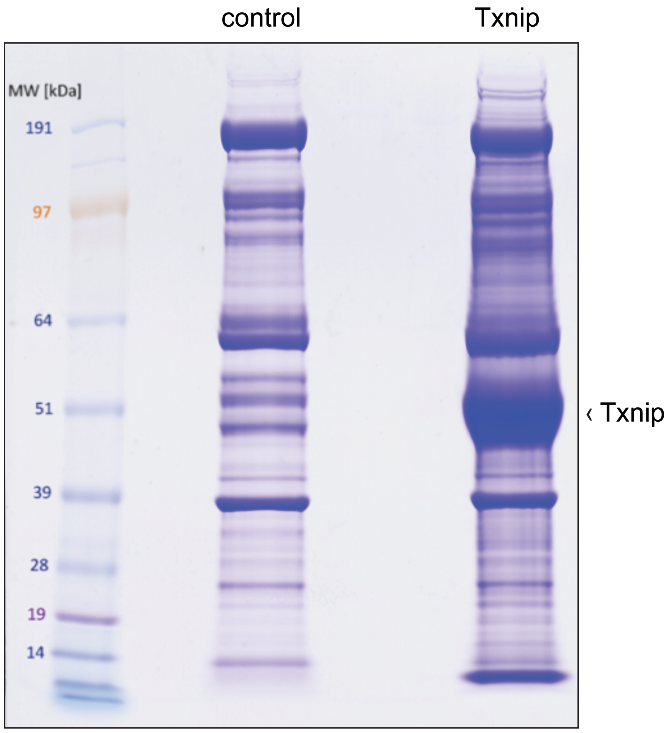

Supplementary Figure S2. Txnip affinity chromatography and SDS-PAGE. Affinity chromatography was performed to purify Txnip, and potential protein-protein interaction partners, from HEK293F cells stably transduced with SF:Txnip or an empty control vector. Eluates were subjected to SDS-PAGE, Coomassie-stained bands were excised and analyzed by mass spectrometry.
