# Supplementary material for: Thioredoxin-interacting protein regulates protein disulfide isomerases and endoplasmic reticulum stress
Source: EMBO Mol Med. 2014 May 19;6(6):732–43. doi: 10.15252/emmm.201302561 (PMC4203352; doi:10.15252/emmm.201302561)
Supplement: Supplementary file 3 — Supplementary Figure S3 [file emmm0006-0732-sd3.pdf]

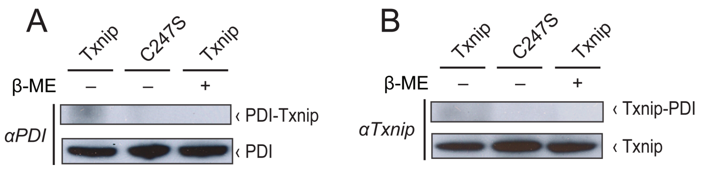

Supplementary Figure S3. Txnip interacts with endogenous PDI. HEK293TN cells were transfected with Txnip or Txnip C247S mutant plasmids. Txnip-PDI complexes were resolved from free **A.** PDI and **B.** Txnip by non-reducing SDS-PAGE following NEM free sulfhydryl alkylation.
