# Supplementary material for: Thioredoxin-interacting protein regulates protein disulfide isomerases and endoplasmic reticulum stress
Source: EMBO Mol Med. 2014 May 19;6(6):732–43. doi: 10.15252/emmm.201302561 (PMC4203352; doi:10.15252/emmm.201302561)
Supplement: Supplementary file 4 — Supplementary Figure S4 [file emmm0006-0732-sd4.pdf]

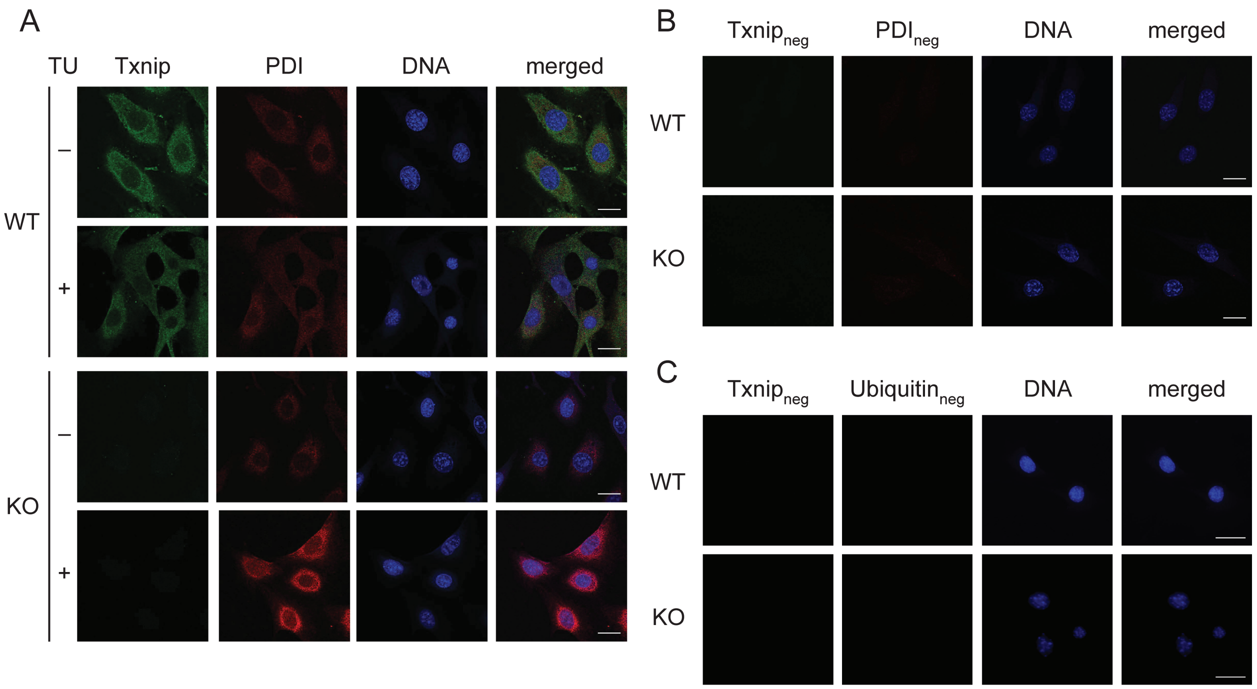

Supplementary Figure S4. Txnip deficiency increases PDI expression. Mouse embryonic fibroblasts from wildtype (WT) and Txnip-null (KO) mice were treated with vehicle or tunicamycin (1 μg/ml) for 6 h. Cells were subsequently fixed, permeablized and stained for indicated proteins. Scale bar = 25 μm. **A.** Protein levels of Txnip and PDI visualized by immunofluorescence under confocal microscopy with **B.** negative controls, probed with secondary antibodies only. **C.** Negative controls of WT and KO cells from Fig. 4A, probed with secondary antibodies only.
