# Supplementary material for: Thioredoxin-interacting protein regulates protein disulfide isomerases and endoplasmic reticulum stress
Source: EMBO Mol Med. 2014 May 19;6(6):732–43. doi: 10.15252/emmm.201302561 (PMC4203352; doi:10.15252/emmm.201302561)
Supplement: Supplementary file 5 — Supplementary Figure S5 [file emmm0006-0732-sd5.pdf]

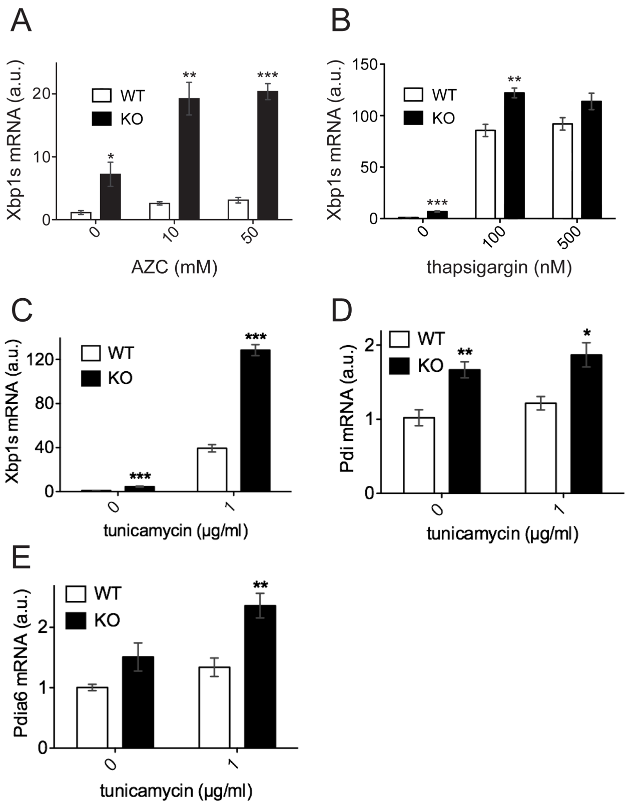

Supplementary Figure S5. Txnip regulates Xbp1s *in vitro*. Relative transcript levels of Xbp1s measured by qPCR normalized to 18S in serum-starved mouse embryonic fibroblasts (MEFs) from wildtype (WT) and Txnip-null (KO) mice treated with increasing concentrations of **A.** L-azetidine carboxylic acid (n = 4) for 2 h, * p = 0.02, ** p = 0.0013, *** p = 0.0003 vs. WT **B.** thapsigargin (n = 4) for 2 h, ** p = 0.003, *** p = 0.0001 vs. WT, and **C.** tunicamycin (n = 4) for 2 h, 0 µg/ml: *** p = 0.0003, 1 µg/ml: *** p = 0.000006 vs. WT. **D.** Relative transcript levels of Pdi, * p = 0.013, ** p = 0.006 vs. WT and **E.** Pdia6 measured by qPCR normalized to 18S in serum-starved MEFs from WT and KO mice treated with tunicamycin (n =4) for 2 h, ** p = 0.007 vs. WT.
