# Supplementary material for: Thioredoxin-interacting protein regulates protein disulfide isomerases and endoplasmic reticulum stress
Source: EMBO Mol Med. 2014 May 19;6(6):732–43. doi: 10.15252/emmm.201302561 (PMC4203352; doi:10.15252/emmm.201302561)
Supplement: Supplementary file 6 — Supplementary Figure S6 [file emmm0006-0732-sd6.pdf]

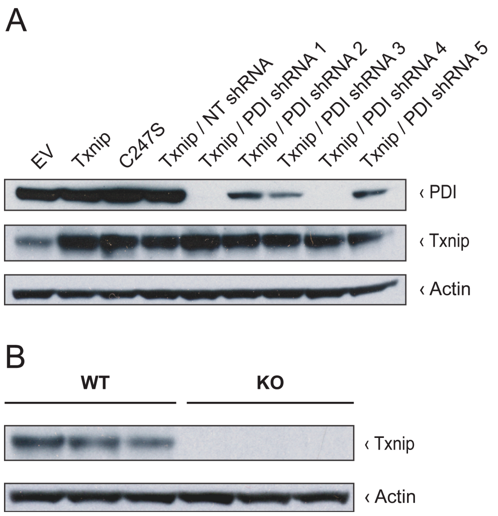

Supplementary Figure S6. Txnip cell lines. A. Protein levels of PDI and Txnip measured by Western analyses in 3T3-L1 fibroblasts stably transduced to overexpress Txnip and to knock down PDI. **B.** Protein levels of Txnip measured by Western analyses in WT and Txnip-KO MEFs.
