# Supplementary material for: Thioredoxin-interacting protein regulates protein disulfide isomerases and endoplasmic reticulum stress
Source: EMBO Mol Med. 2014 May 19;6(6):732–43. doi: 10.15252/emmm.201302561 (PMC4203352; doi:10.15252/emmm.201302561)
Supplement: Supplementary file 7 — Supplementary Figure S7 [file emmm0006-0732-sd7.pdf]

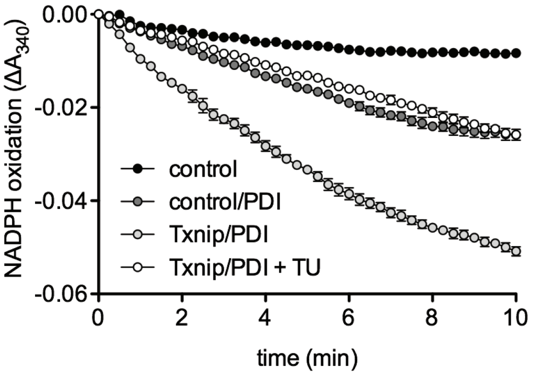

Supplementary Figure S7. ER stress affects PDI activity *in vitro*. HEK293TN cells were transfected with the indicated plasmids in the presence or absence of tunicamycin (1 μg/ml x 2 h) and lysates were used to perform enzymatic activity assays. PDI activity was measured using a coupled insulin reduction assay (n = 3).
