# Supplementary material for: Thioredoxin-interacting protein regulates protein disulfide isomerases and endoplasmic reticulum stress
Source: EMBO Mol Med. 2014 May 19;6(6):732–43. doi: 10.15252/emmm.201302561 (PMC4203352; doi:10.15252/emmm.201302561)
Supplement: Supplementary file 8 — Supplementary Figure S8 [file emmm0006-0732-sd8.pdf]

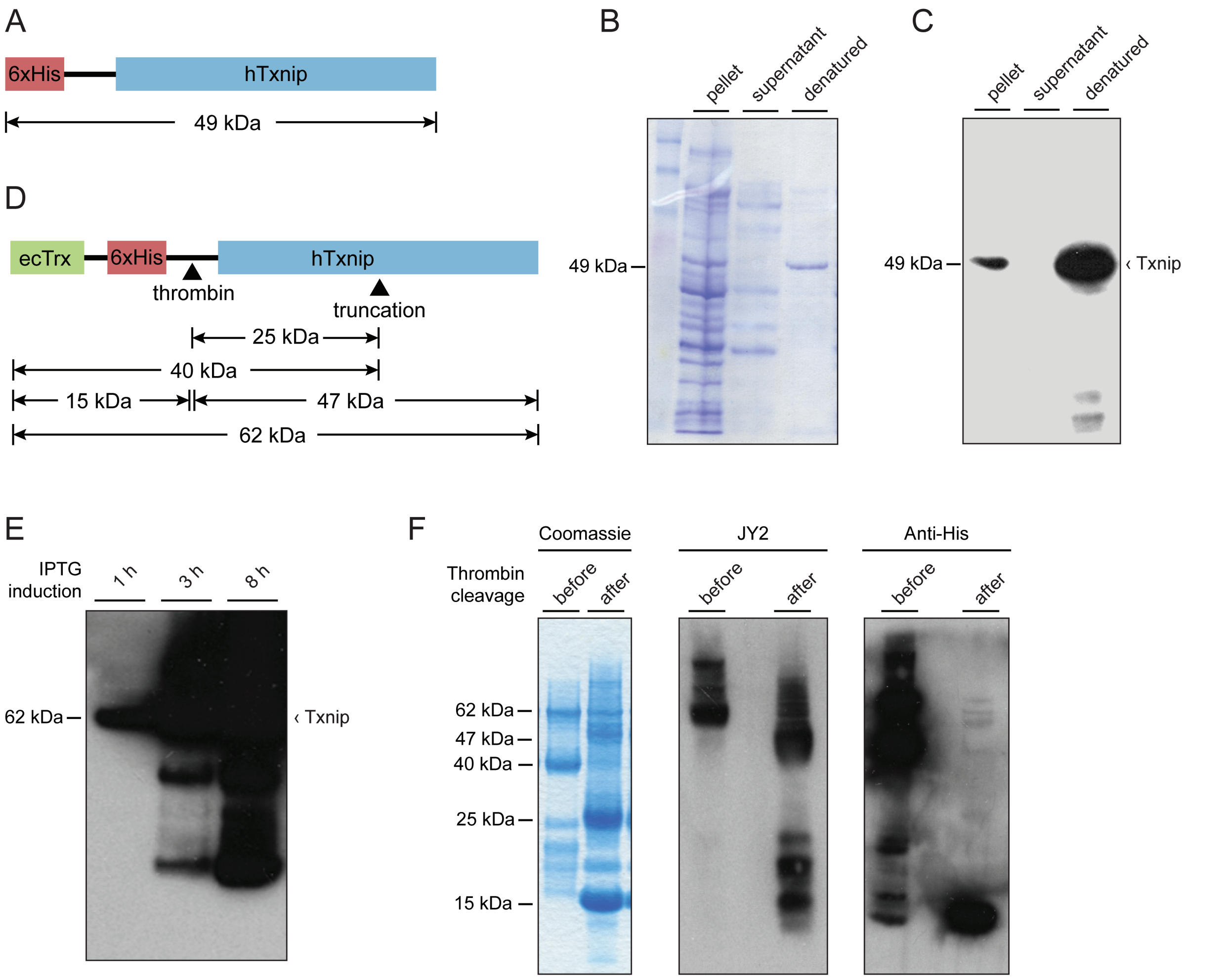

Supplementary Figure S8. Txnip purification. A. Schematic representation of recombinant human Txnip (hTxnip) with an N-terminal 6xHistidine-tag (6xHis) in pTrcHis TOPO (Invitrogen). **B.** Coomassie staining and **C.** Western analysis of protein lysates from E. coli overexpressing hTxnip (pellet), from supernatant of E. coli cultures (supernatant), and purified Txnip in denaturing conditions. Recombinant hTxnip protein was purified in denatured condition; however, solubility was limited in the supernatant fraction. **D.** Schematic representation of recombinant human Txnip with an N-terminal 6xHis-tag in fusion with E. coli Thioredoxin (ecTrx) in pET-32a(+) (Novagen). This system was used to enhance the production

and solubility of hTxnip. **E.** Western analysis of protein levels of ecTrx-6xHis-hTxnip fusion protein in E. coli lysates after increasing durations of induction with Isopropyl β-D-1-thiogalactopyranoside (IPTG). **F.** Coomassie staining of ec-Trx-6xHis-hTxnip fusion protein after Ni-NTA-column purification and subsequent SDS-PAGE before and after thrombin cleavage with corresponding Western analyses using an anti-Txnip (JY2) and an anti-His-tag antibody. Although hTxnip protein was successfully purified in native condition with this method, there were multiple bands in Coomassie staining, indicating significant amounts of contamination by other proteins.
