# Supplementary material for: Thioredoxin-interacting protein regulates protein disulfide isomerases and endoplasmic reticulum stress
Source: EMBO Mol Med. 2014 May 19;6(6):732–43. doi: 10.15252/emmm.201302561 (PMC4203352; doi:10.15252/emmm.201302561)
Supplement: Supplementary file 9 — Supplementary Figure S9 [file emmm0006-0732-sd9.pdf]

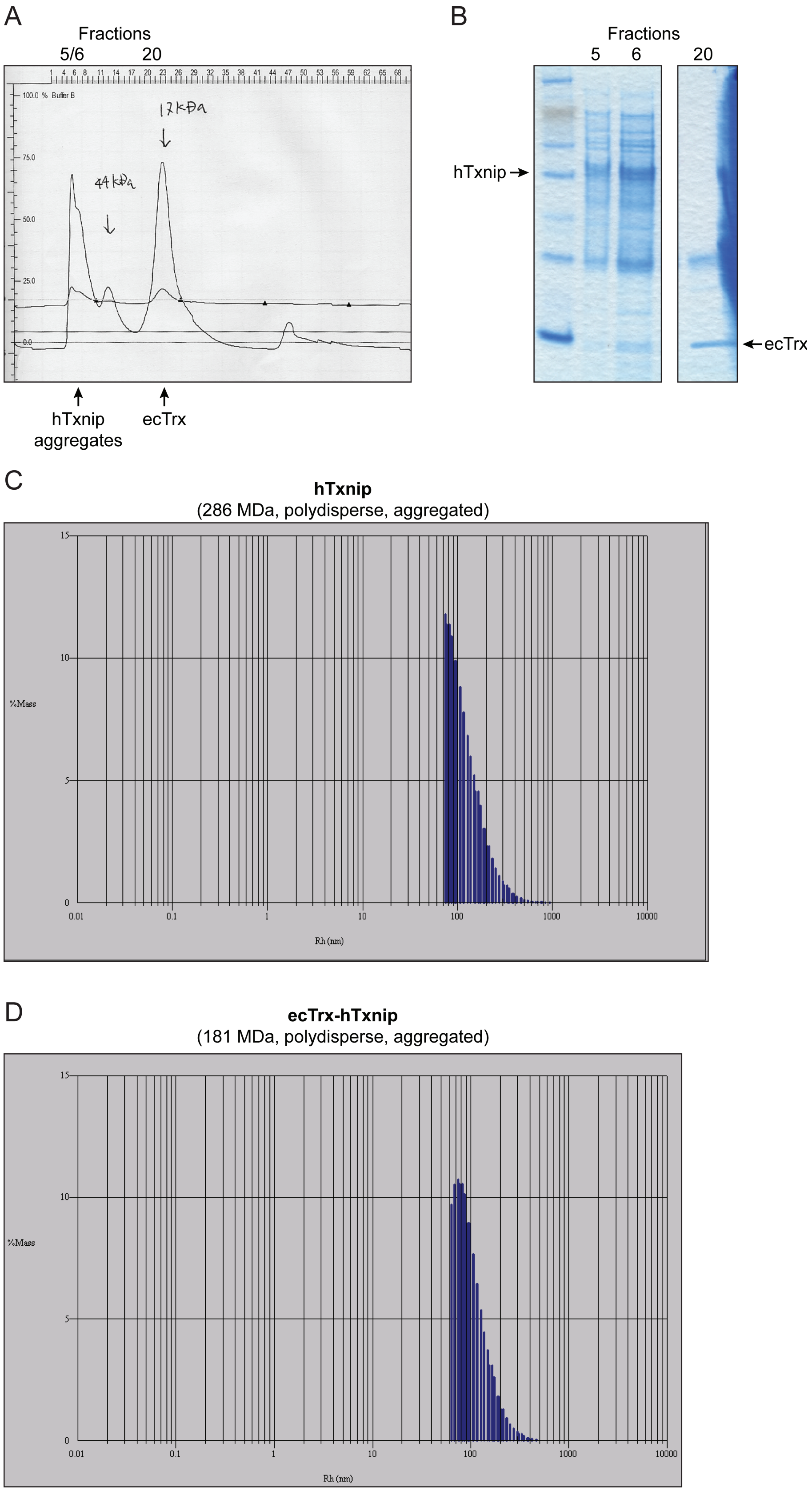
A
Fractions
5/6
20
17kDa
44kDa
100.0 % Buffer B
75.0
50.0
25.0
0.0
hTxnip aggregates
ecTrx
B
Fractions
5
6
20
hTxnip
ecTrx
C
hTxnip
(286 MDa, polydisperse, aggregated)
%Mass
Rh (nm)
0.01
0.1
1
10
100
1000
10000
D
ecTrx-hTxnip
(181 MDa, polydisperse, aggregated)
%Mass
Rh (nm)

Supplementary Figure S9. Txnip aggregation. A. Chromatogram of ecTrx-6xHis-hTxnip fusion protein after thrombin cleavage and gel filtration high performance liquid chromatography (HPLC). **B.** Coomassie staining of SDS-PAGE of fractions 5/6 (hTxnip) and 20 (ecTrx). Dynamic light scattering (DLS) demonstrated that the particle size of purified **C.** 6xHis-hTxnip and **D.** ecTrx-6xHis-hTxnip proteins was over 286 MDa and 181 MDa even with maximal usage of DTT. Thus, after purifying hTxnip protein by HPLC, hTxnip (fraction 5 and 6) formed protein aggregates.
