# Supplementary material for: Thioredoxin-interacting protein regulates protein disulfide isomerases and endoplasmic reticulum stress
Source: EMBO Mol Med. 2014 May 19;6(6):732–43. doi: 10.15252/emmm.201302561 (PMC4203352; doi:10.15252/emmm.201302561)
Supplement: Supplementary file 10 — Supplementary Materials and Methods [file emmm0006-0732-sd10.pdf]

SUPPLEMENTARY MATERIALS AND METHODS

Txnip-thioredoxin complex trapping by free-sulfhydryl alkylation

Free-slufhydryls alkylation was performed as described previously (Chutkow & Lee, 2012). Briefly, 293TN cells were transfected with Txnip wild type and mutant constructs. 24 hours after transfection, cells were washed twice in ice-cold PBS, and proteins were precipitated with ice-cold 0.54 M trichloroacetate in PBS. Lysates were incubated on ice for 10 min, then centrifuged at 9000 x g for 10 min at 4 °C. The pellets were washed with acetone, then centrifuged at 9000 x g for 10 min at 4 °C. The resulting pellets were briefly air-dried on ice then dissolved in NEM-labeling buffer (62.5mMTris,pH6.8, 1% SDS, 25mM NEM) and incubated at 4 °C with end-over-end rotation for 18 h, then incubated at 37 °C for 10 min. After adding nonreducing sample loading buffer and sonicating, the labeled proteins were subjected to SDS-PAGE and Western analysis.

Txnip purification

Human Txnip (XM_002093.5) transcript was subcloned into pTrcHis TOPO TA expression vector (Invitrogen) and pET-32a(+) vector (Novagen), and transformed into E. coli. Human Txnip recombinant protein expression was induced with IPTG, and purified using the ProBond purification system (Invitrogen) for isolating His-tagged recombinant protein.

pET32b contains a cleavable His-tag and E. coli thioredoxin; thrombin was used to cleave E.coli thioredoxin from h-Txnip (Supportin Information Fig S6A). Cleavage of the fusion protein was verified by Coomassie Blue Stain and Western Blot (Supporting Information Fig S6F), with a

band at 47 kDa for Txnip and a band for E.coli-Trx and His-tag at 15 kDa. Then, the protein was purified using high performance liquid chromatography (HPLC). For size exclusion chromatography, thrombin was dialyzed against 20 mM Tris-HCl, pH 8, 50 mM NaCl buffer. Then, chromatography was performed using HPLC equipment. Purified protein was subjected to gel filtration on a HiPrep 16/30 Sephacryl 5-100 high resolution column (GE Healthcare) at room temperature. Buffer A containing 150 mM NaCl was used as eluent at a flow-rate of 1.0 ml/min. Protein was monitored by measuring absorbance at 280 nm with fractions of 2 ml. The column was calibrated with a mixture of thyroglobulin (670 kDa), γ-globulin (158 kDa), ovalbumin (44 kDa) and cytochrome c (17 kDa).

Purified protein was resuspended in the same buffer solution, 150 mM NaCl, 20 mM Tris-HCl, pH 8.0, and dynamic light scattering (DLS) was performed. A sample of 100 μl (1 mg/ml) was passed through a Millex-HV 0.45 μm pore size filter (Millipore). Then the protein was placed into a quartz cuvette and measured by a laser spectroscatter 201 (Berlin, Germany) for 90 s at 20 °C. Supporting Information Figure S7C shows the results obtained for the %Mass vs Rh (nm) spectra. These results show that Txnip protein formed aggregates, as we measured high molecular weight (> MegaDalton) of the sample. We calculated a radius of 127.9 nm with polydispersion of 66.69 nm, which also demonstrates aggregation of the protein.
