# Supplementary figures and images for: Thioredoxin-interacting protein regulates protein disulfide isomerases and endoplasmic reticulum stress
Source: EMBO Mol Med. 2014 May 19;6(6):732–43. doi: 10.15252/emmm.201302561 (PMC4203352; doi:10.15252/emmm.201302561)

SOURCE DATA

FIGURE 1A

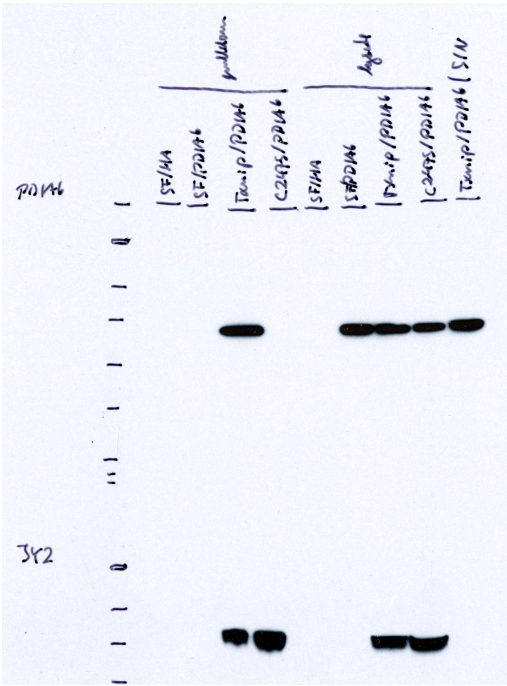

Figure 1B

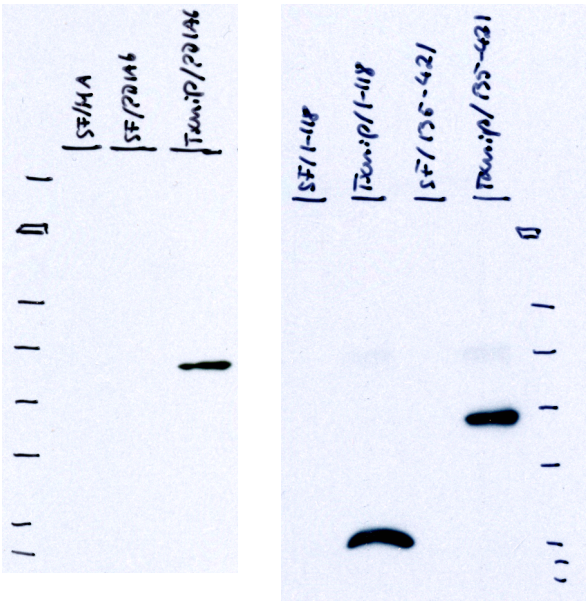

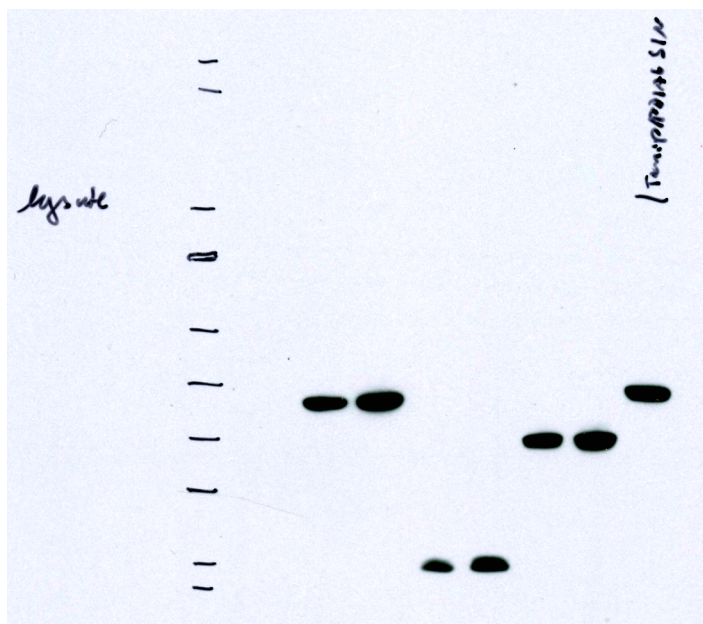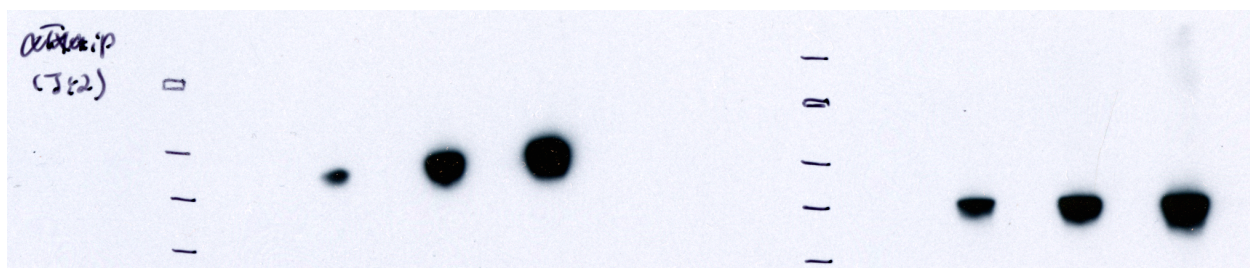

Figure 1C

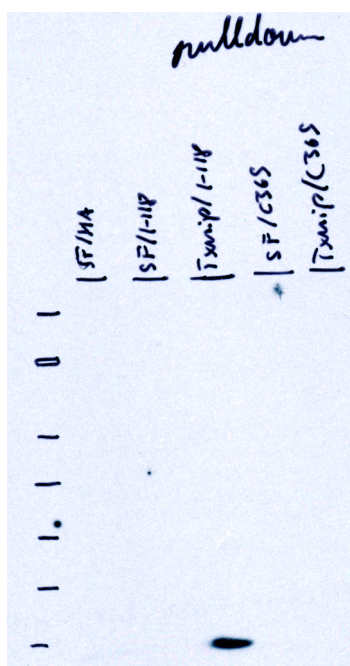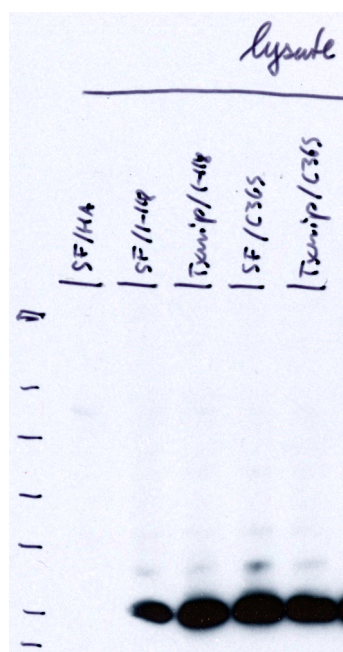

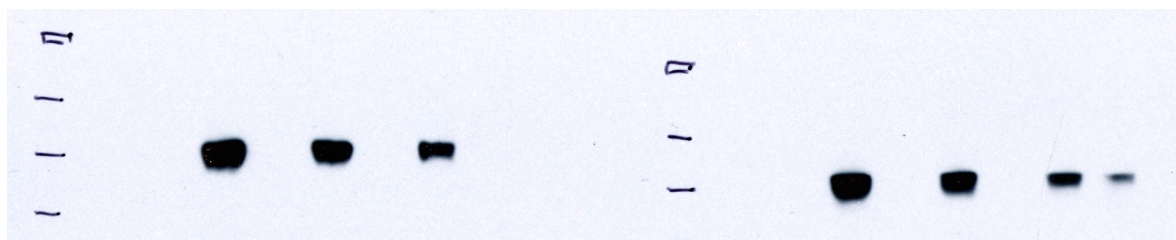

**Figure 1D**

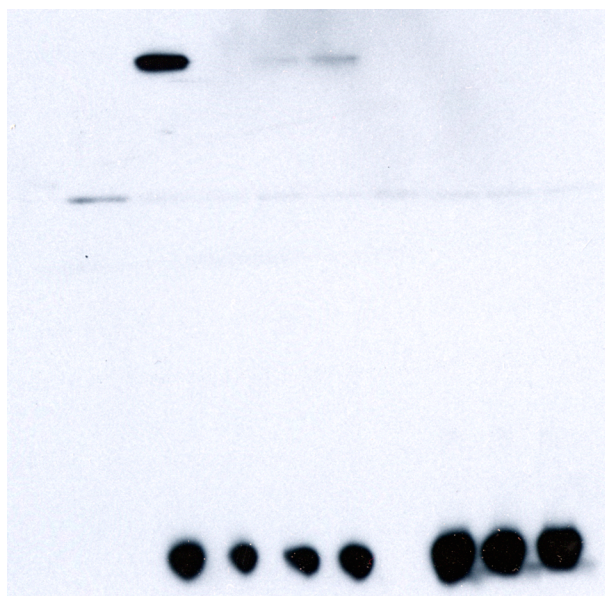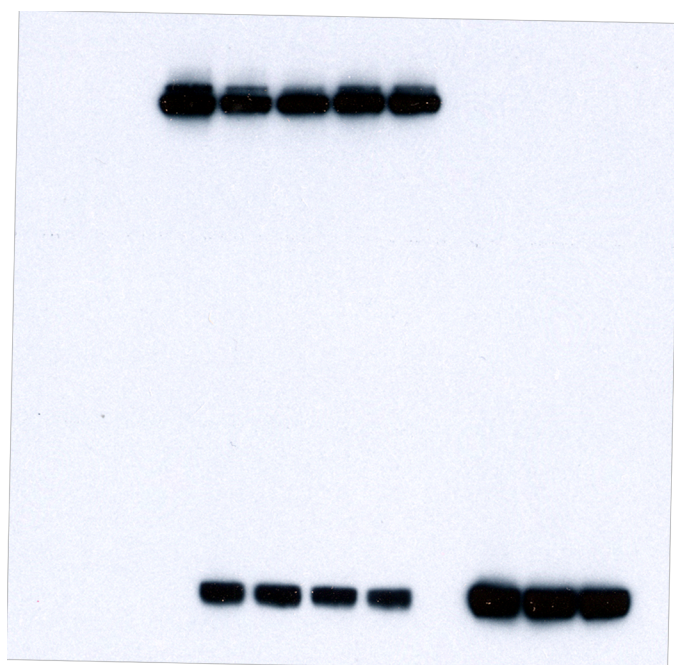

Supplement: Supplementary file 13 — Source Data for Figure 1 A B C D [file emmm0006-0732-sd13.pdf]

SOURCE DATA

Figure 2A

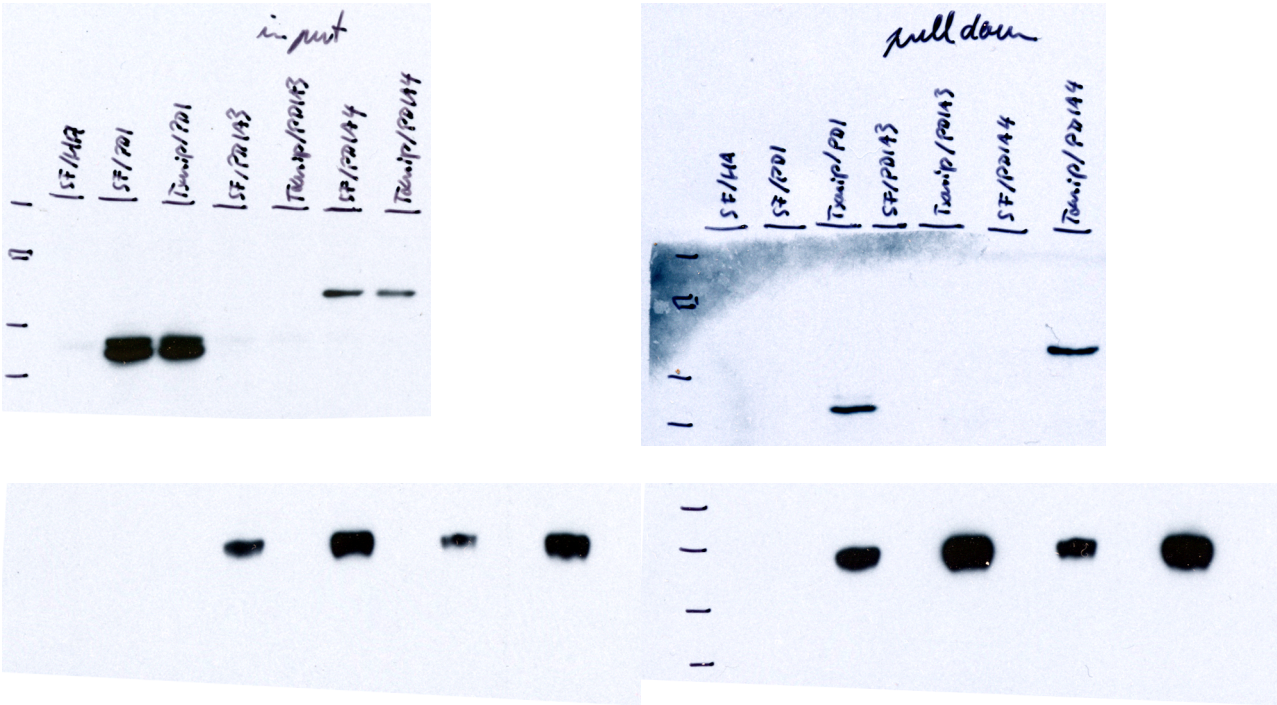

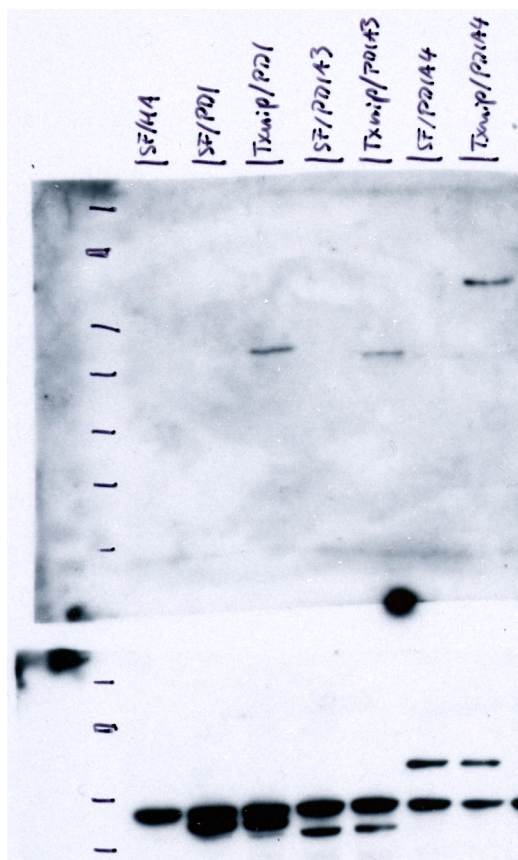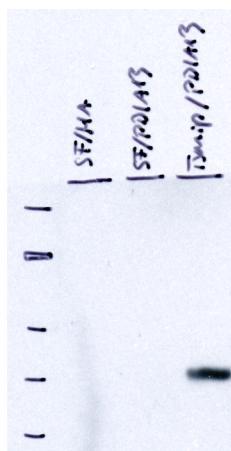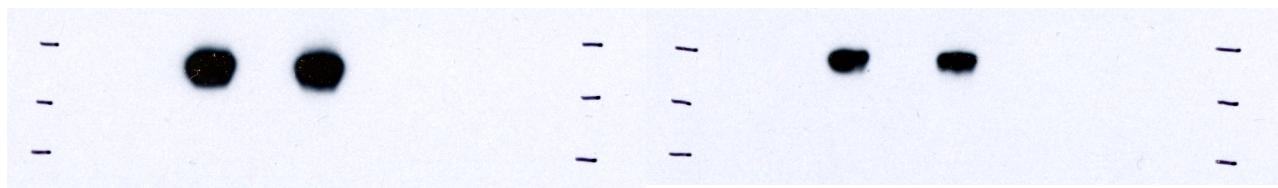

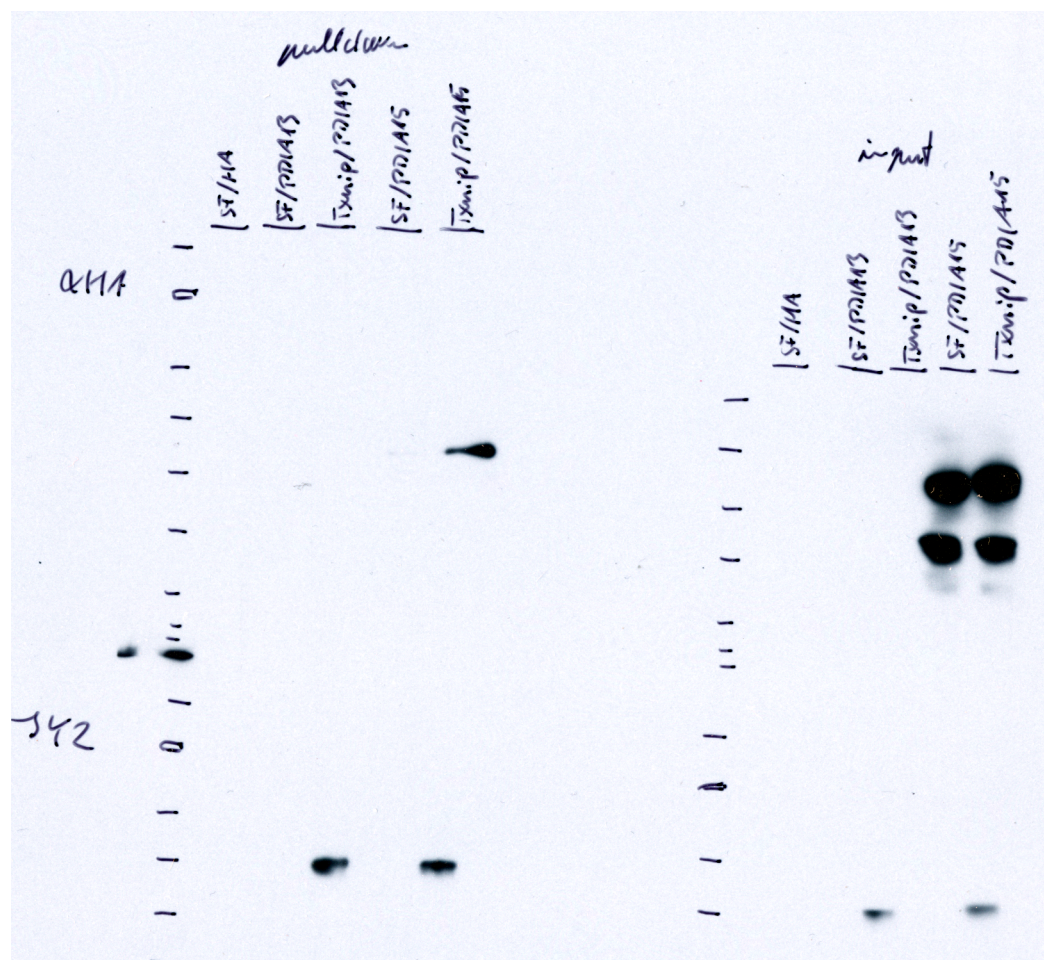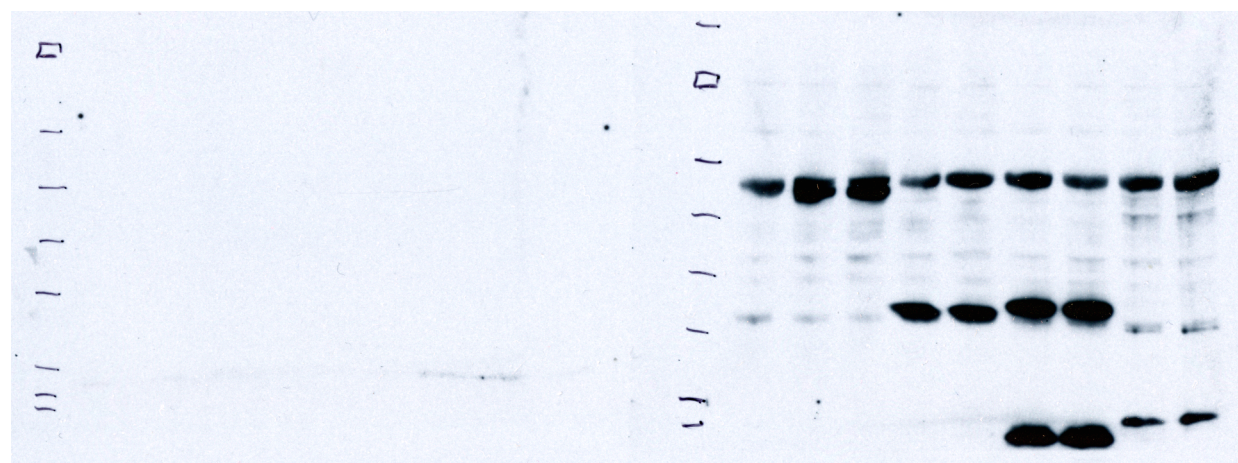

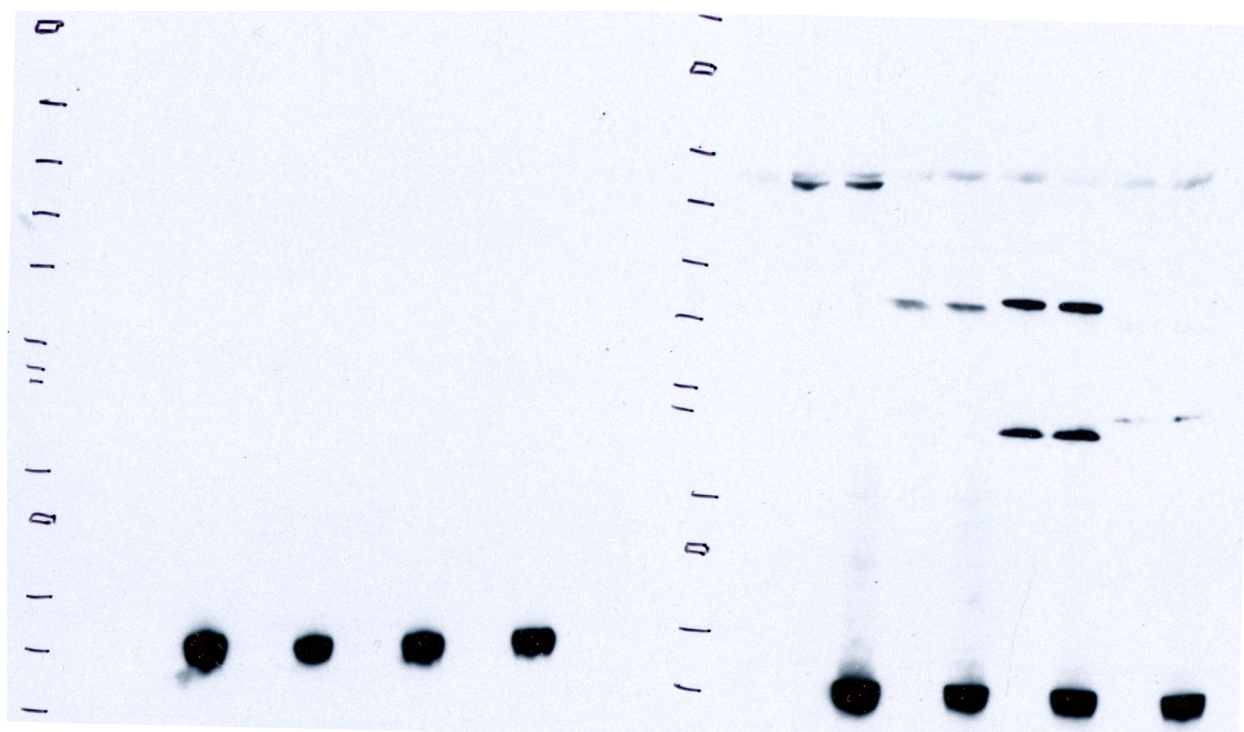

**Figure 2B**

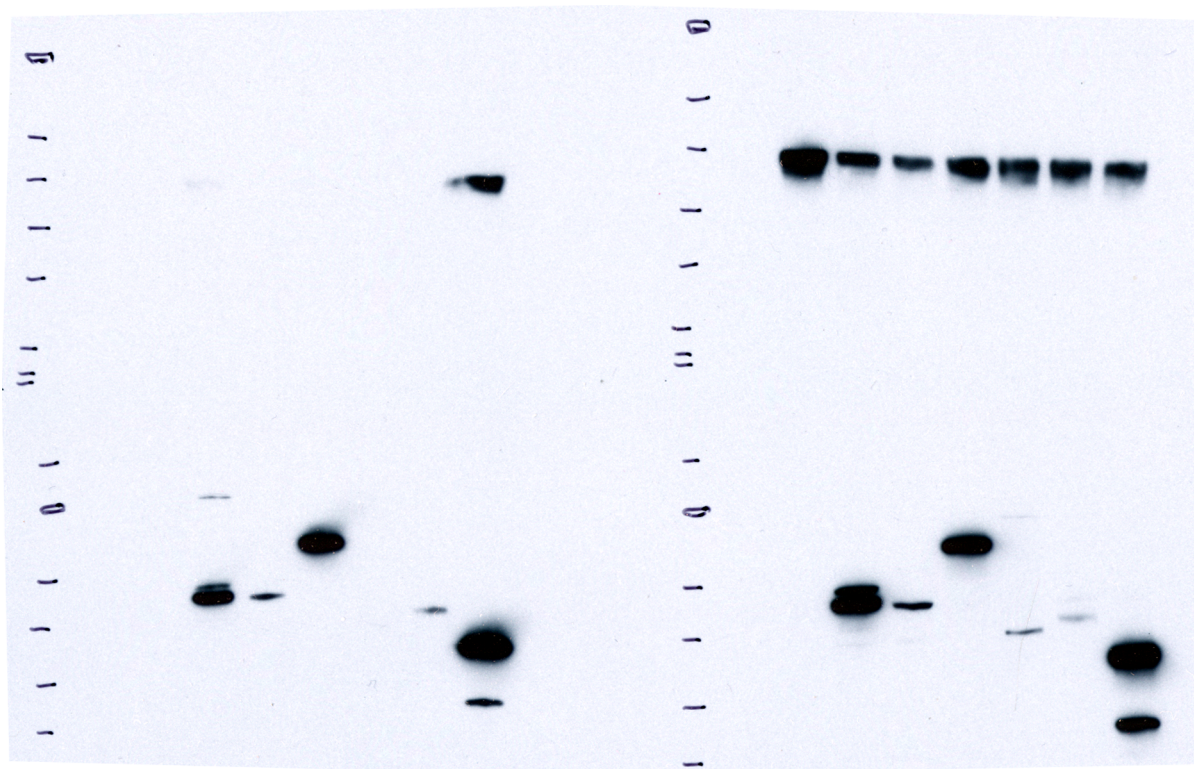

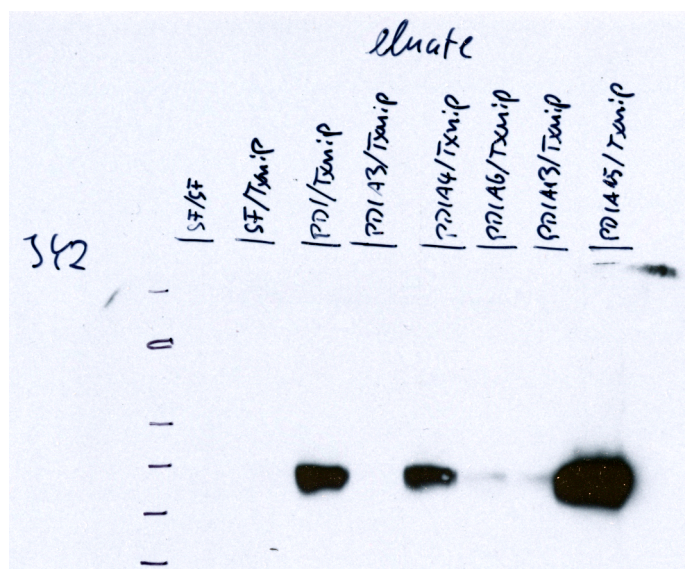

Figure 2C

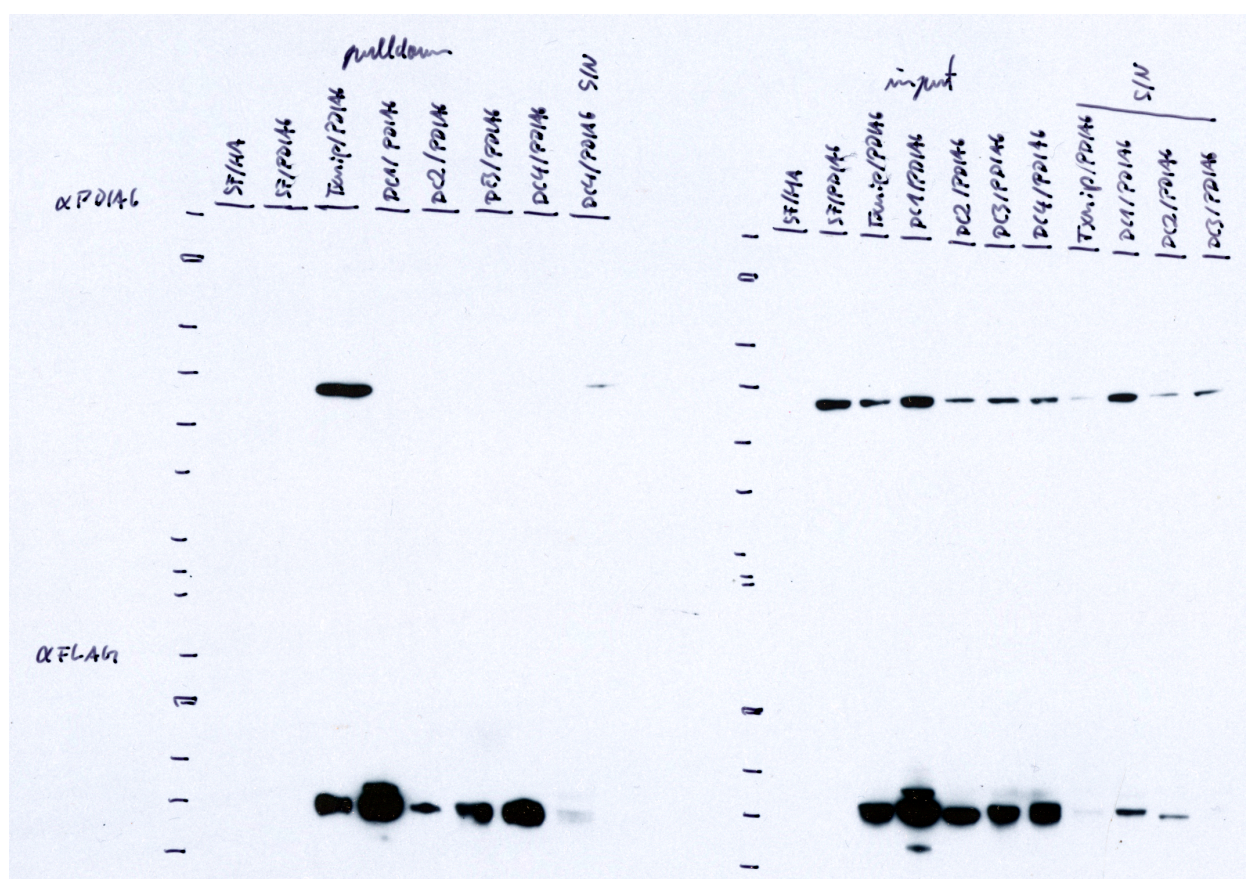

Supplement: Supplementary file 14 — Source Data for Figure 2 A B C [file emmm0006-0732-sd14.pdf]

SOURCE DATA

Figure 3A

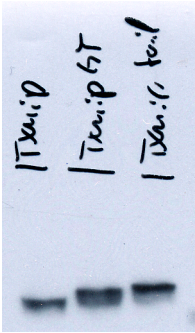

Figure 3B

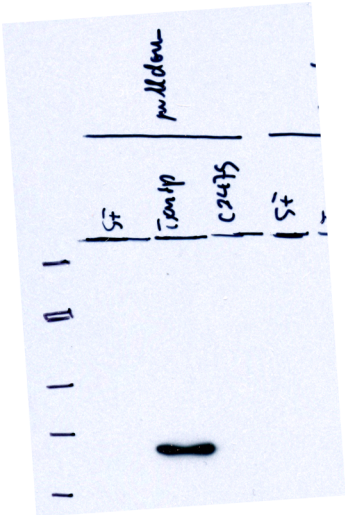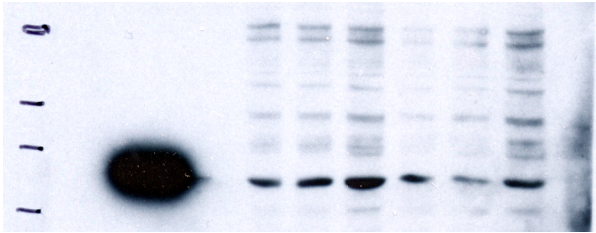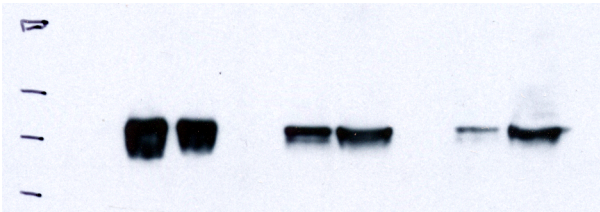

Supplement: Supplementary file 15 — Source Data for Figure 3 A B [file emmm0006-0732-sd15.pdf]

SOURCE DATA

Figure 6E

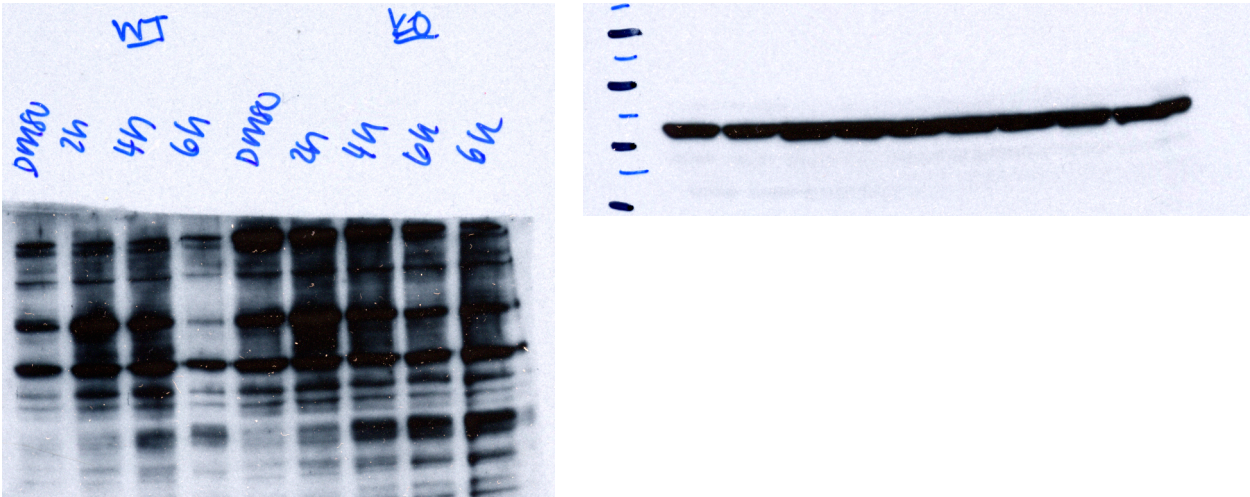

Figure 6F

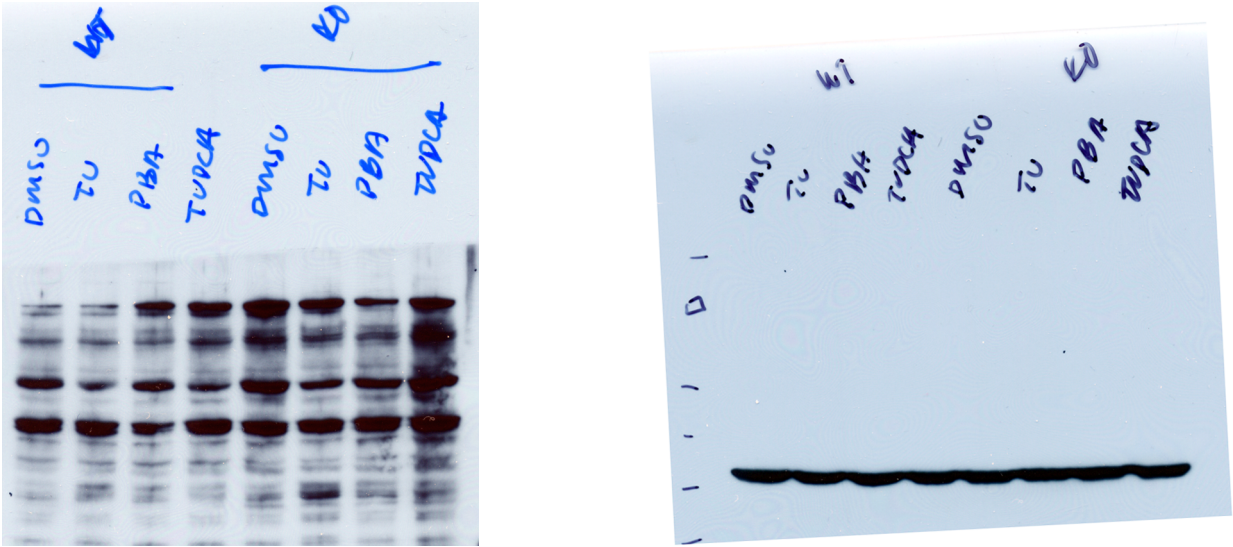

Figure 6G

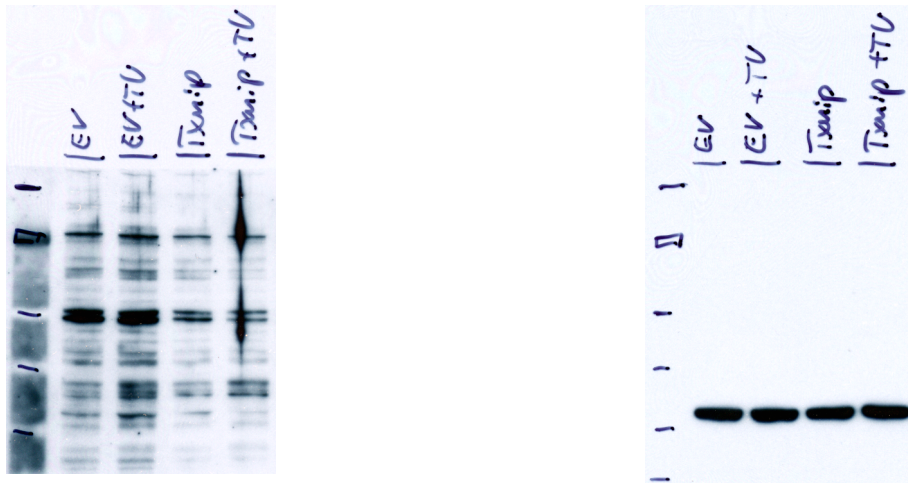

Supplement: Supplementary file 16 — Source Data for Figure 6 E F G [file emmm0006-0732-sd16.pdf]

## SOURCE DATA

**Figure 7E**

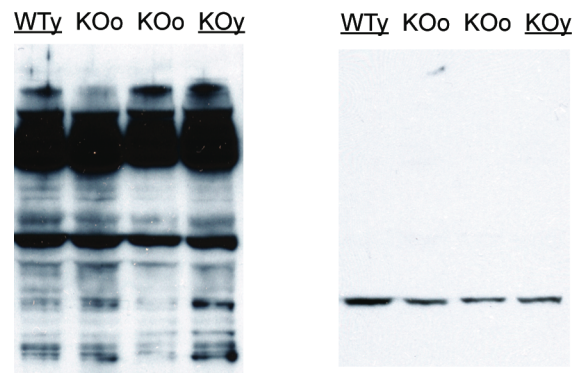

Supplement: Supplementary file 17 — Source Data for Figure 7 E [file emmm0006-0732-sd17.pdf]
